# Abstract of the Proceedings of the Buffalo Medical Association

**Published:** 1856-09

**Authors:** Sanford B. Hunt


ART. IV.—*Abstract of the Proceedings of the Buffalo Medical Association.*

TUESDAY EVENING, Aug. 5, 1856.

The association met.

Present—Drs. Eastman, Miner, Whitney, Newman, Hawley, Lemon, Hunt, Baker, Dayton, Hamilton, Hutchins, Wilcox, Howell, Wyckoff, and Lay.

The President being absent, Dr. Baker was called to the chair.

The minutes of the preceding meeting were read and approved.

*Prof. Hamilton* moved that Dr. Rogers be invited to sit with the association until such time as he could become a member of the Erie County Medical Society.

Dr. D. Devening was admitted to membership on motion of Dr. Newman.

Dr. Whitehead was admitted to membership on motion of Dr. Hamilton.

*Prof. Hamilton* presented a specimen of the middle finger which had been torn off by machinery. Its peculiar feature was, that in being torn out it removed with it one of the flexor tendons to the length of about fifteen inches. On the fourth day there was slight tenderness along the track of the tendon as far as the elbow, but this immediately subsided, and no inconvenience had followed. That a long narrow wound, such as this must have produced, was closed with no inflammation, is probably owing to the entire exclusion of air. Not so much that air is an irritant, but the fact that air enters implies a want of coaptation of the parts.

*Dr. Howell* reported a case of solar exhaustion, occurring on the 12th of July. The patient was a little girl, aged 10 years, who had been playing in the sun during the day. When Dr. H. arrived she had been lying insensible for about an hour and a-half. She was pale, pulseless, and profoundly insensible; respiration slow but not labored; and her appearance did not indicate congestion of the brain. She recovered in half to three-quarters of an hour under the use of stimulants, and revulsives applied externally. Dr. H. regarded it as peculiarly a case of solar exhaustion, and not of *coup de soleil*, coupling the idea of congestion of the brain with the latter condition.

He also regarded it as confirmatory of the theory advanced by Prof. Hunt, in the June number of the Buffalo Medical Journal, as to humidity being the active element of causation. The 12th of July was not a very warm day, but it was exhaustive; the dew-point was very high. There was rain on the 11th and 13th, but none on the 12th. The air of the 12th was humid, and the sun obscured somewhat by haze. The condition of the patient, too, was evidently one of profound exhaustion of the nervous system, and not of cerebral congestion.

*Dr. Baker* then read the following account of a

### *Case of Rupture of the Liver.*

Pauline Duboile, a French girl, eleven years of age, on her way to school, the morning of the 18th ult., was knocked down and run over by a [illegible]tcher's wagon.

Immediately after the accident she got up and walked with a staggerin gait, some ninety feet (twice crossing the street) and sank exhausted to the ground.

She was carried home by her mother (about eighty rods) and died fifteen minutes after her arrival, and a half hour after the accident. She spoke but once after the receipt of the injury. As her mother was lifting her to her arms she cried out, "Oh mother, you hurt me."

At the request of Coroner Nott I examined the body ten hours after death. The surface was unusually pale for one dying suddenly and in full health, and the sugillation along the back and other depending portions of the body, was remarkably slight. The only marks of external violence were a bruise as though made by the cork of a horse shoe, on the inner edge of left patella and an ecchymosed spot a half an inch in diameter, near the middle of the 9th rib on the left side. There was apparently a depression of the ribs over the heart—but this was not distinct.

The absence of external marks of violence indicated internal injury as the cause of death, and the paleness of the skin induced a belief that there had been internal hæmorrhage.

Opening the cavity of the chest the lungs were found healthy and uninjured. The heart appeared healthy, occupying its normal position, empty of blood and uninjured. Dissecting below the diaphragm the peritoneum was found distended with blood. Laying open this membrane I removed nearly two quarts of dark fluid blood; and, carefully dissecting the liver from its attachments, removed it from its place, when it was found ruptured through the entire length of the right lobe, a distance of six or seven inches, and so nearly through its entire substance, as to hang together only by a few shreds on its under surface.

The stomach was partially distended with the food taken at breakfast, but unaffected. The pancreas showed no signs of injury. The spleen was ruptured for some two inches along the middle of its lower edge and surface.

The intestines appeared healthy and uninjured. The brain was not examined, as the head had escaped injury.

The principal point of interest in this case to myself was, the extensive rupture of the liver with so little external injury.

Dr. Baker also reported, verbally, the case of a man who was kicked by a horse over the pubis, on Monday, July 28th. The shock was not very severe, and though feeling ill, he was able to walk about up to Thursday morning, July 31st. He died quite suddenly Thursday P. M. An autopsy revealed a gangrenous state of the peritoneum generally, with an abundant effusion of caco-plastic lymph between the convolutions of the intestines. Probably perforation existed, but the intestines were so easily torn that it could not be located.

Dr. Eastman (President) here took the chair.

*Dr. Whitney* mentioned the following incident, on the authority of an intelligent layman who witnessed it:

A hog was run over by the cars in this city, the other day, and its heart torn from the body and thrown some distance on the pavement. It pulsated violently for ten or twelve beats after striking the pavement, and then continued more moderate pulsations for from three to five minutes, before it finally ceased. He regarded this as a very long period for the pulsation of the heart of a warm-blooded animal out of the body.

*Dr. Wilcox* mentioned, that some years since he was called, by the coroner, to examine the body of a woman of dissipated habits, who had suffered much from ague and had an enlarged spleen. She was kicked by a horse in the afternoon, kept about during the day, but felt faint and prostrated at night, and died the next morning. The examination revealed a rupture of the spleen, of about one and a-half inches in length. A large amount of blood had escaped into the peritoneal cavity, which had coagulated, leaving a firm clot which would weigh some three pounds. The serum separating was so abundant that it was drawn off by a trocar, before opening the abdomen.

Dr. Wilcox also furnished another case, of an Irishman, who was struck upon the right side by a long lever, which broke when he was prying upon it. He felt faint for a little time, but rallied again; but finally died in four hours from the time of the accident. He found a rupture of the liver of a stellated form, and contused. But little blood was effused.

*Dr. Baker* stated that he was called, Aug. 2, to make a *post-mortem* examination of a man who was killed by a blow from a sweep. He was struck on the left side of the head and killed instantly. Dr. B. found a fracture extending from the left ear around through the occipital bone to the right ear. The fracture was most extensive on the *right* side. There was also another fracture running perpendicularly through the left parietal bone.

On the inquiry of the President as to prevalent diseases, no epidemic was reported, and only sporadic cases of summer diseases.

And the association then adjourned.

SANFORD B. HUNT, Secretary.

